# Supplementary material for: A SERS Study of Charge Transfer Process in Au Nanorod–MBA@Cu2O Assemblies: Effect of Length to Diameter Ratio of Au Nanorods
Source: Nanomaterials (Basel). 2021 Mar 29;11(4):867. doi: 10.3390/nano11040867 (PMC8066000; doi:10.3390/nano11040867)
Supplement: Supplementary file 1 [file nanomaterials-11-00867-s001.pdf]

Supplementary Materials

A SERS Study of Charge Transfer Process in Au Nanorod–MBA@Cu_2O Assemblies: Effect of Length to Diameter Ratio of Au Nanorods

Lin Guo [1], Zhu Mao [2], Sila Jin [3], Lin Zhu [1], Junqi Zhao [1], Bing Zhao [1,*] and Young Mee Jung [3,*]

[1] State Key Laboratory of Supramolecular Structure and Materials, Jilin University, Changchun 130012, China; linguo18@mails.jlu.edu.cn (L.G.); zhulin17@mails.jlu.edu.cn (L.Z.); Zhaojq19@mails.jlu.edu.cn (J.Z.)

[2] School of Chemistry and Life Science, Changchun University of Technology, Changchun 130012, China; maozhu@ccut.edu.cn

[3] Department of Chemistry, Institute for Molecular Science and Fusion Technology, Kangwon National University, Chuncheon 24341, Korea; jsira@kangwon.ac.kr

* Correspondence: zhaob@mail.jlu.edu.cn (B.Z.); ymjung@kangwon.ac.kr (Y.M.J.)

Citation: Guo, L.; Mao, Z.; Jin, S.; Zhu, L.; Zhao, J.; Zhao, B.; Jung, Y.M. A SERS Study of Charge Transfer Process in Au Nanorod-MBA@Cu_2O Assemblies: Effect of Length to Diameter Ratio of Au Nanorods. *Nanomaterials* **2021**, *11*, 867. https://doi.org/10.3390/nano11040867

Academic Editor: Ronald Birke

Received: 23 February 2021
Accepted: 24 March 2021
Published: 29 March 2021

Publisher's Note: MDPI stays neutral with regard to jurisdictional claims in published maps and institutional affiliations.

Copyright: © 2021 by the authors. Submitted for possible open access publication under the terms and conditions of the Creative Commons Attribution (CC BY) license (http://creativecommons.org/licenses/by/4.0/).

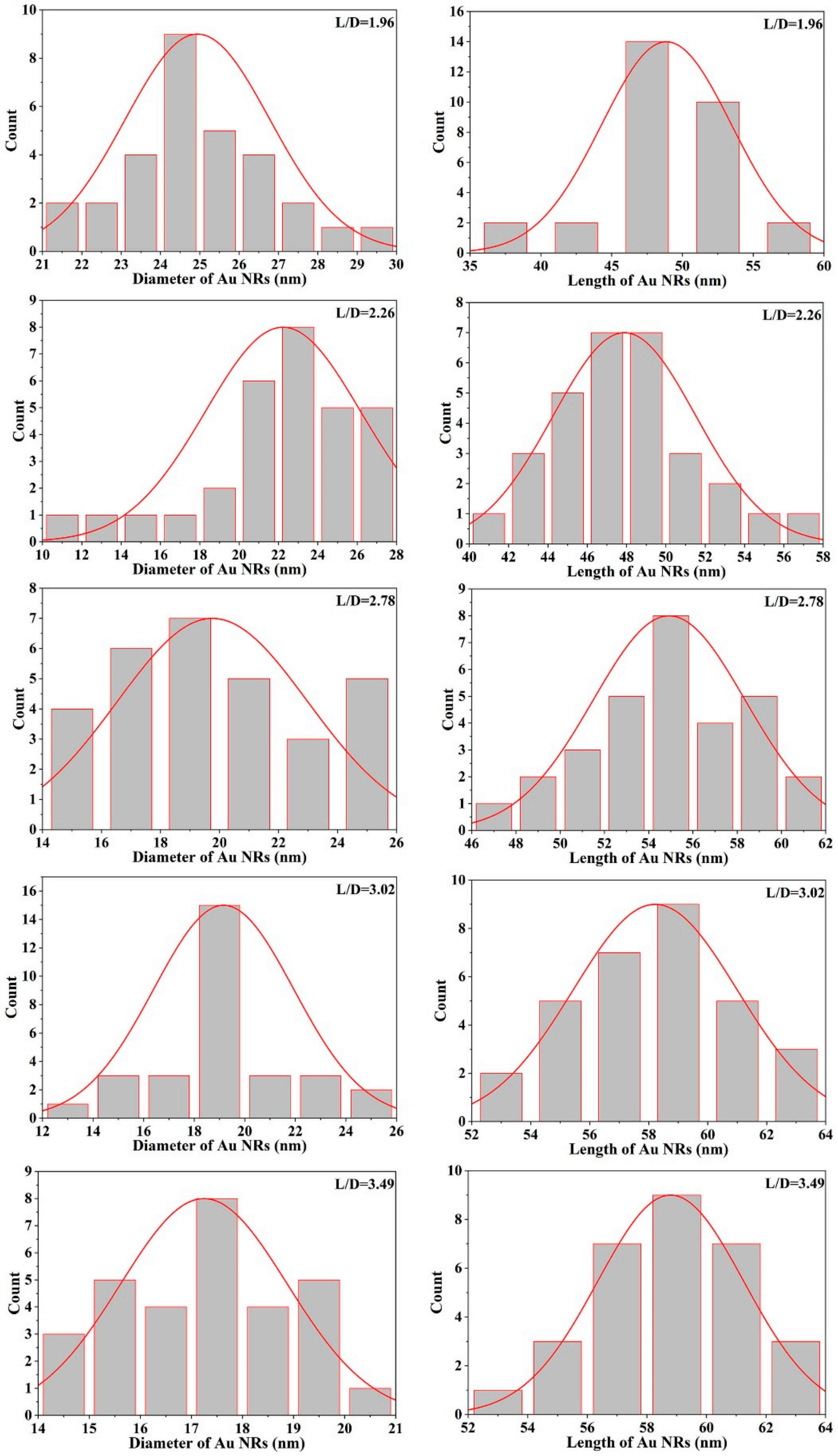

Figure S1. Size distribution of the Au NRs with different L/Ds: 1.96, 2.26, 2.78, 3.02, and 3.49.

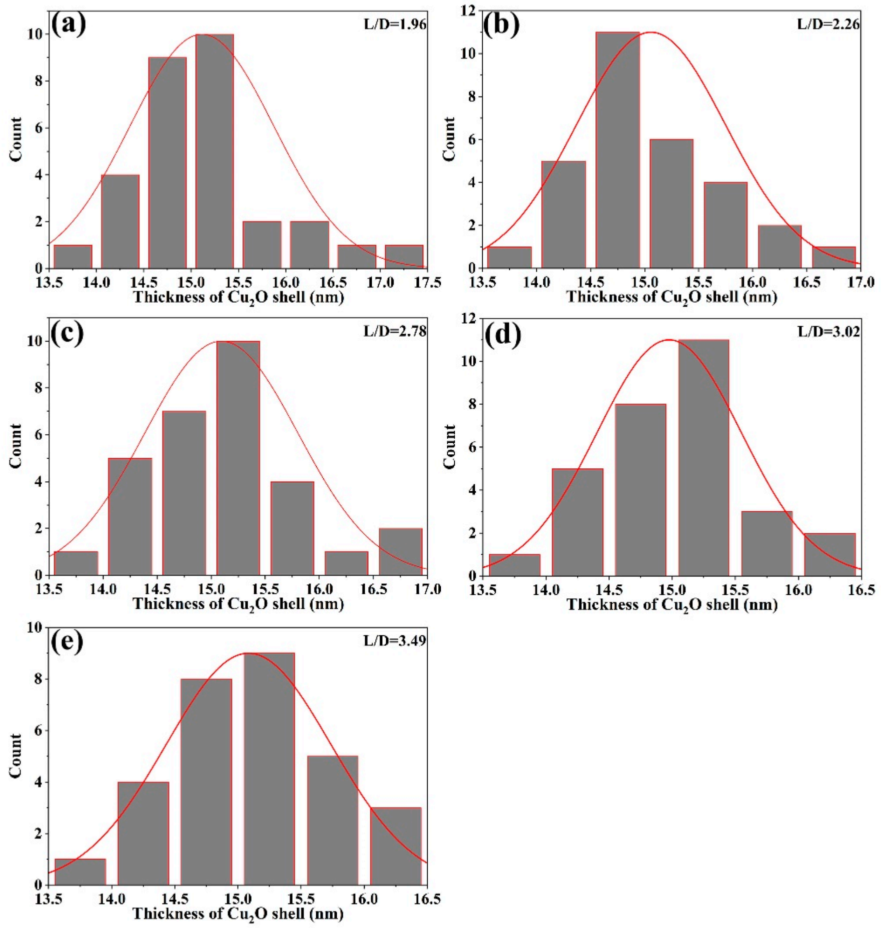

Figure S2. Size distribution of the Cu_2O shell thicknesses with different L/Ds of (a) 1.96, (b) 2.26, (c) 2.78, (d) 3.02, and (e) 3.49.

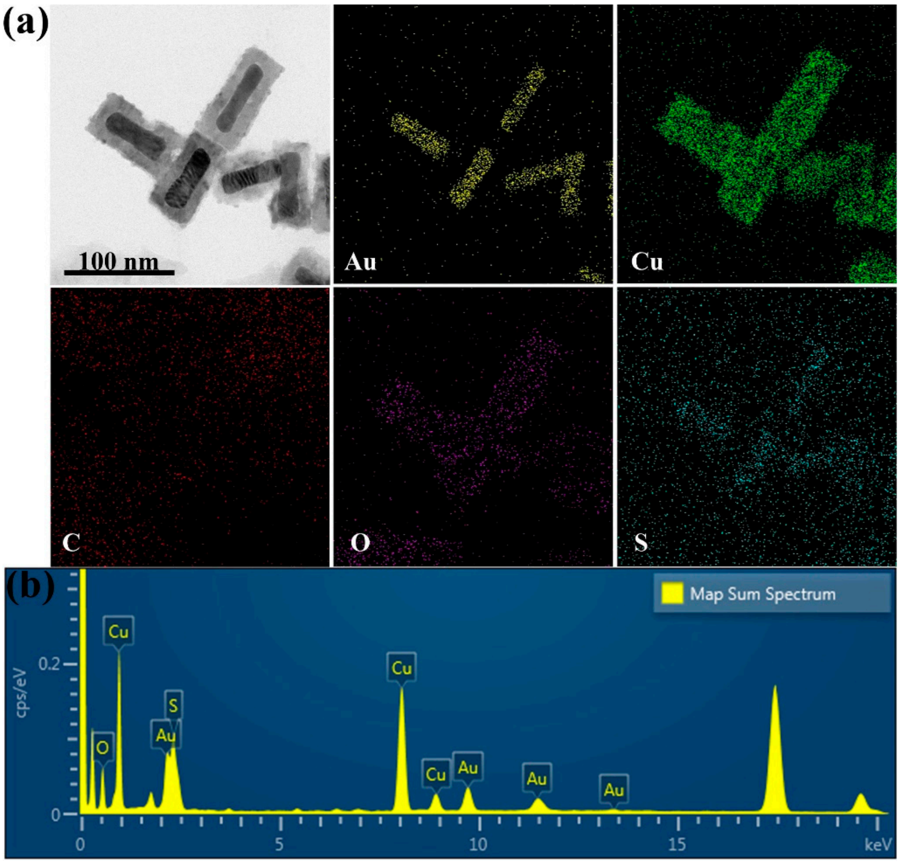

Figure S3. (**a**) Au, O, S, C and Cu elemental mapping of Au NR-MBA@Cu_2O assemblies on a molybdenum grid. (**b**) EDX spectrum of Au NR-MBA@Cu_2O assemblies (L/D = 2.78), which indicates the successful assembly of Au NR-MBA@Cu_2O and the high purity of the assemblies.

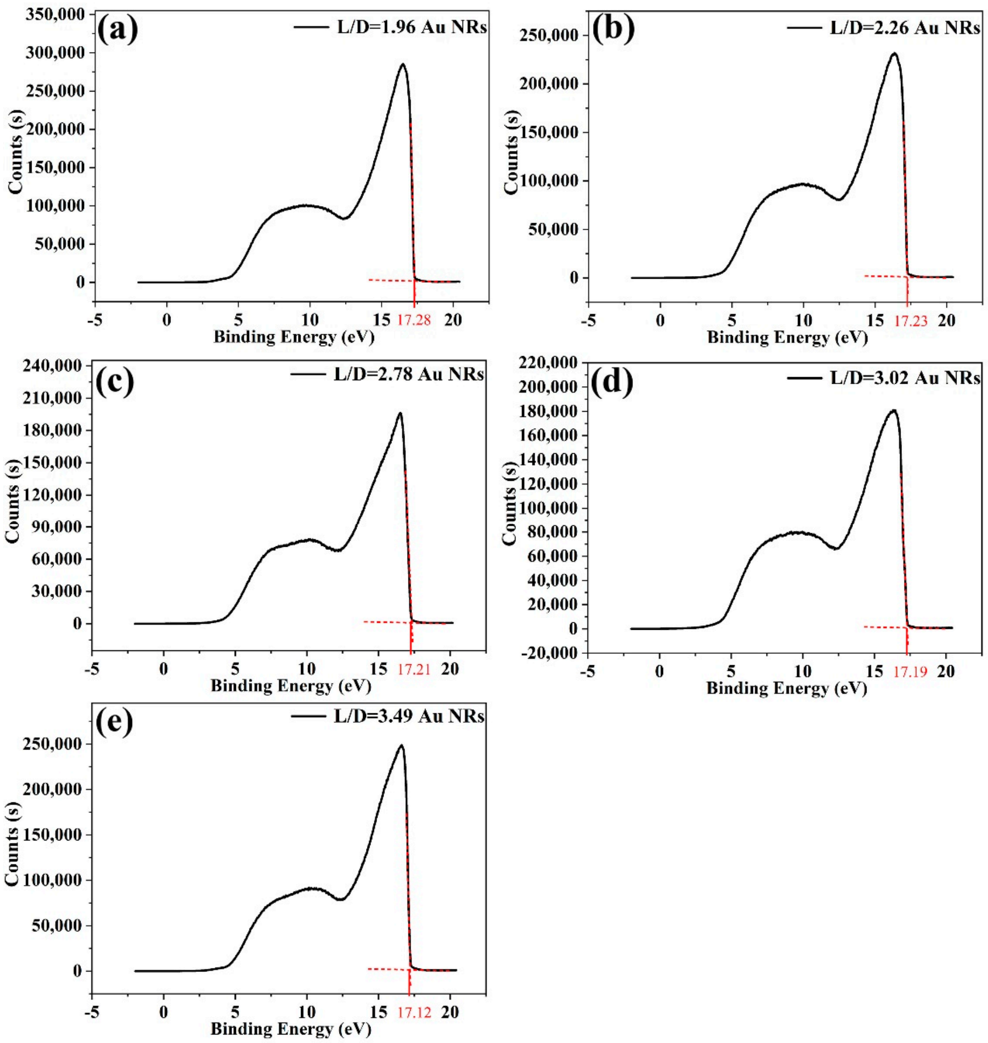

Figure S4. The UPS spectra of Au NRs with different L/Ds: (a) 1.96, (b) 2.26, (c) 2.78, (d) 3.02, and (e) 3.49.

Using the data in Figure S4, the location of the Fermi level of the Au NRs can be calculated from the equation: $W_F = h\nu - \Delta E$. Thus, the Fermi level of the Au NRs with L/Ds of 1.96, 2.26, 2.78, 3.02, and 3.49 respectively correspond to 3.94, 3.99, 4.01, 4.03, and 4.10 eV from the vacuum level.

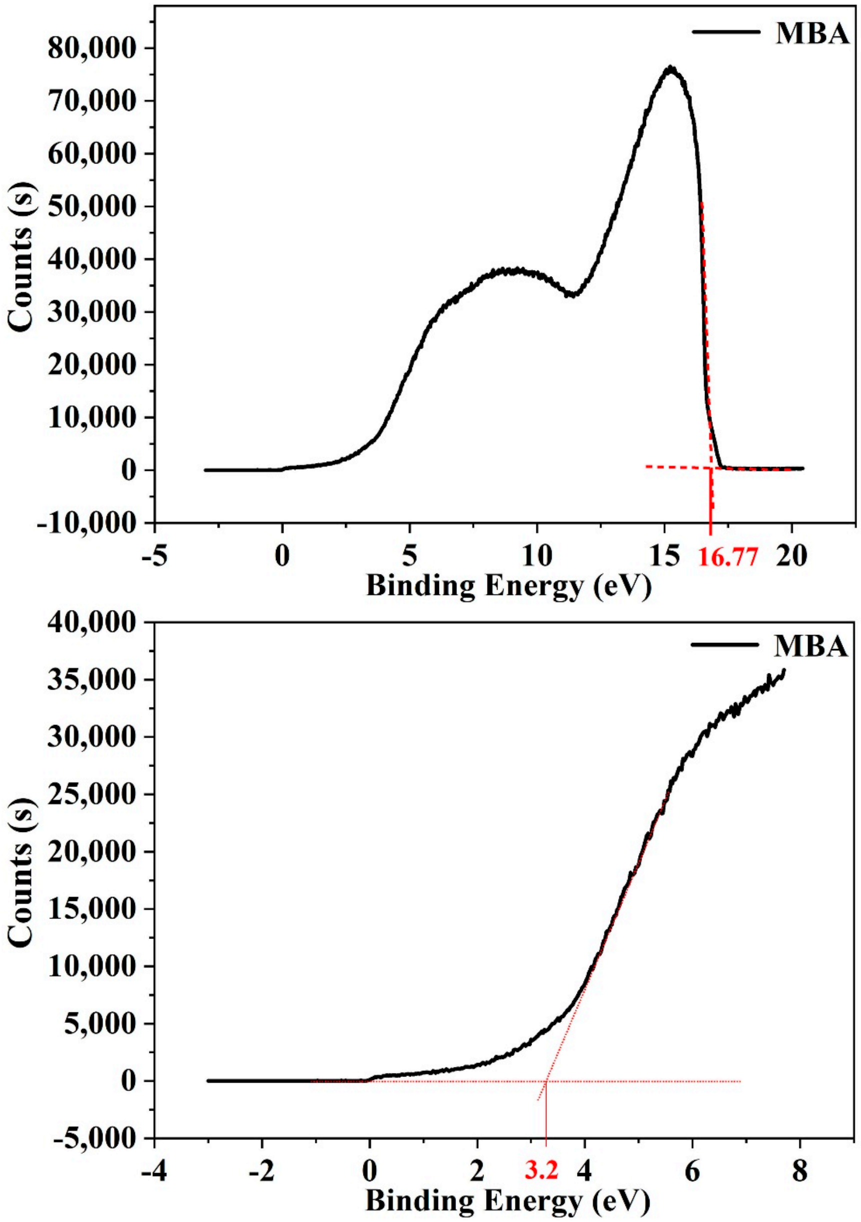

Figure S5. The UPS spectra of MBA.

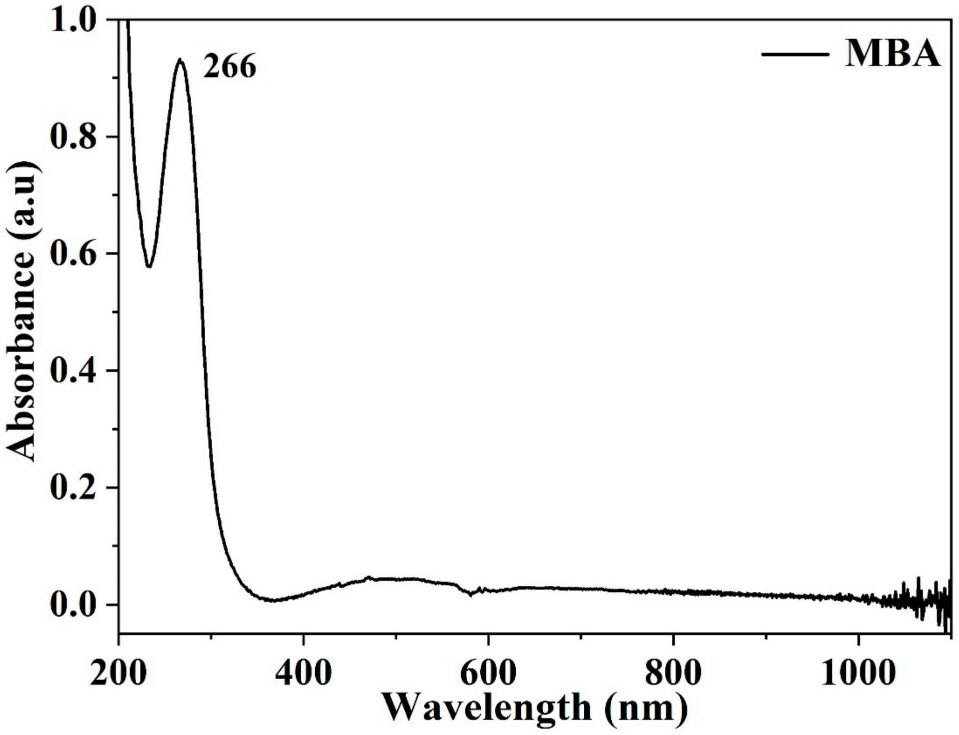

Figure S6. The UV-Vis spectrum of MBA.

As indicated in Figure S5, the work function of MBA is 4.45 eV, and the HOMO of MBA is at 7.65 eV. As shown in Figure S6, the UV-Vis adsorption band is observed at 266 nm. Thus, the HOMO-LUMO band gap is 4.66 eV. The LUMO of MBA is at 2.99 eV.

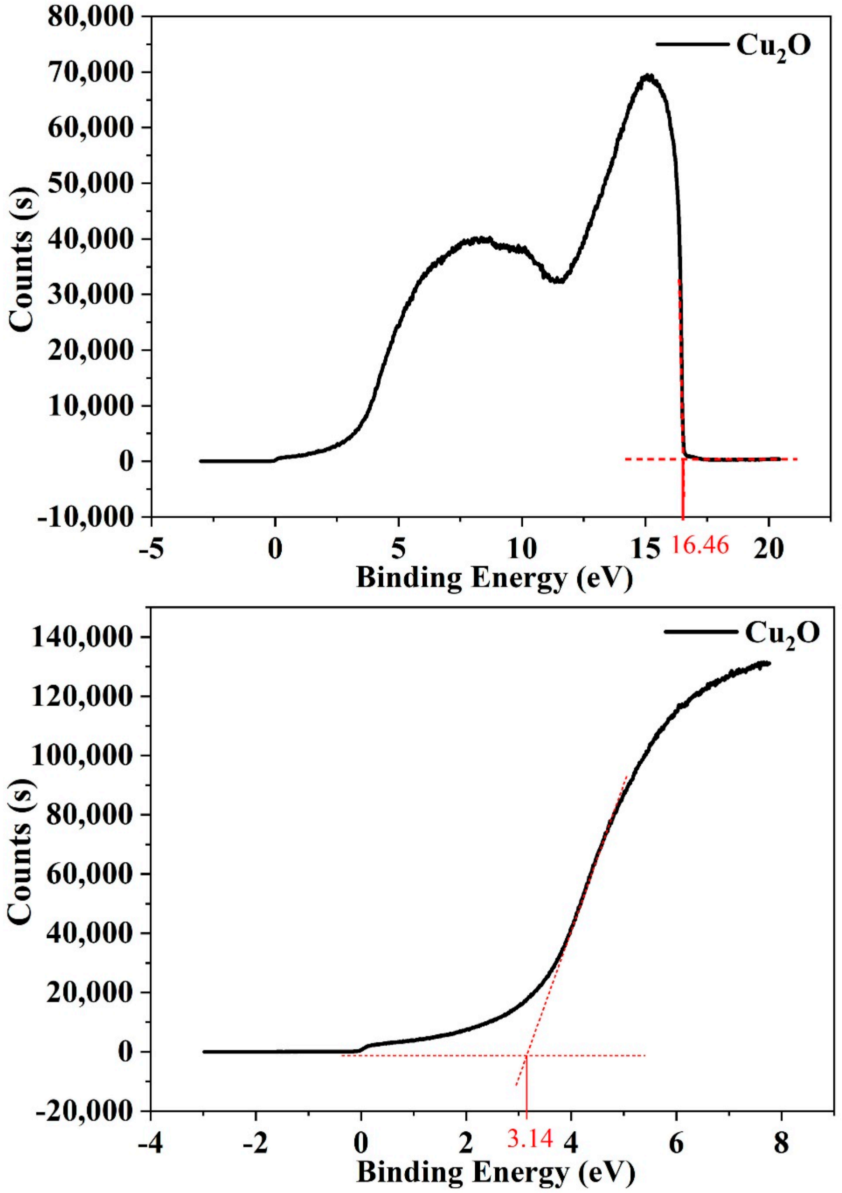

Figure S7. The UPS spectra of Cu_2O.

According to Figure S7, the work function of Cu_2O is 4.76 eV, and the VB of Cu_2O is at 7.9 eV. The band gap of Cu_2O between the VB and CB is 2.2 eV. Thus, the CB of Cu_2O is at 5.7 eV.

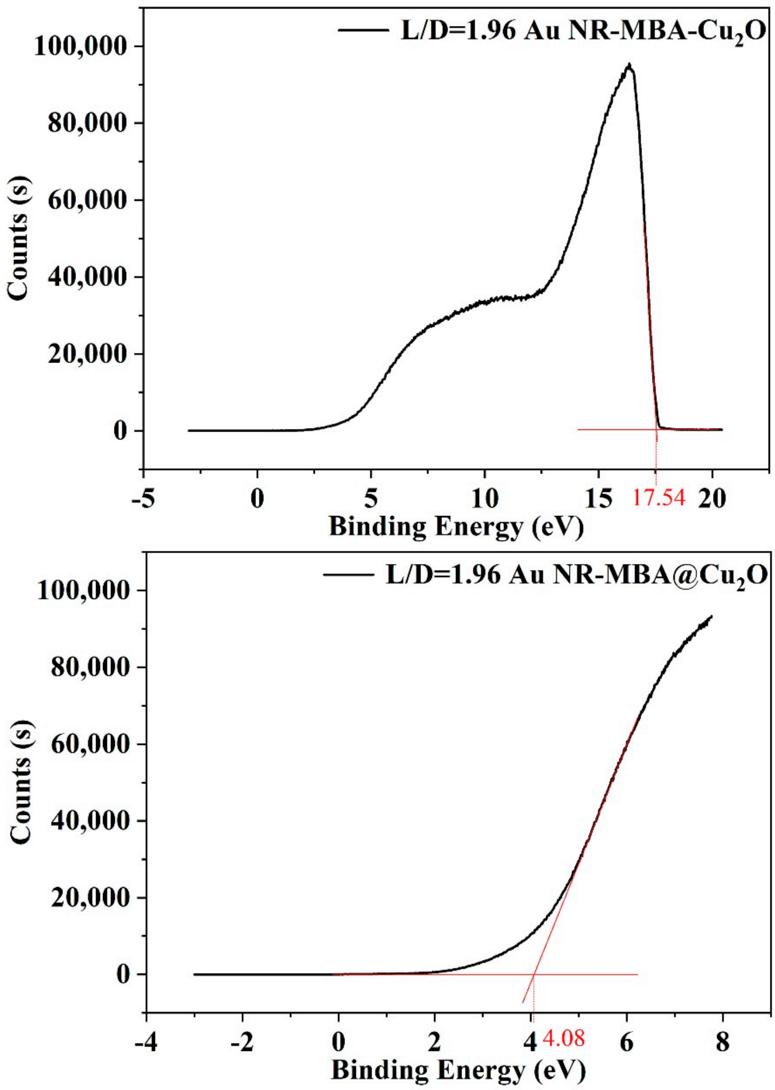

Figure S8. The UPS spectra of Au NR-MBA@Cu_2O with L/D=1.96.

According to Figure S8, the work function of Au NR-MBA@Cu_2O with L/D=1.96 is 3.68 eV, and the VB of Cu_2O is at 7.76 eV. The band gap of Cu_2O between the VB and CB is 2.2 eV. Thus, the CB of Cu_2O is at 5.56 eV.

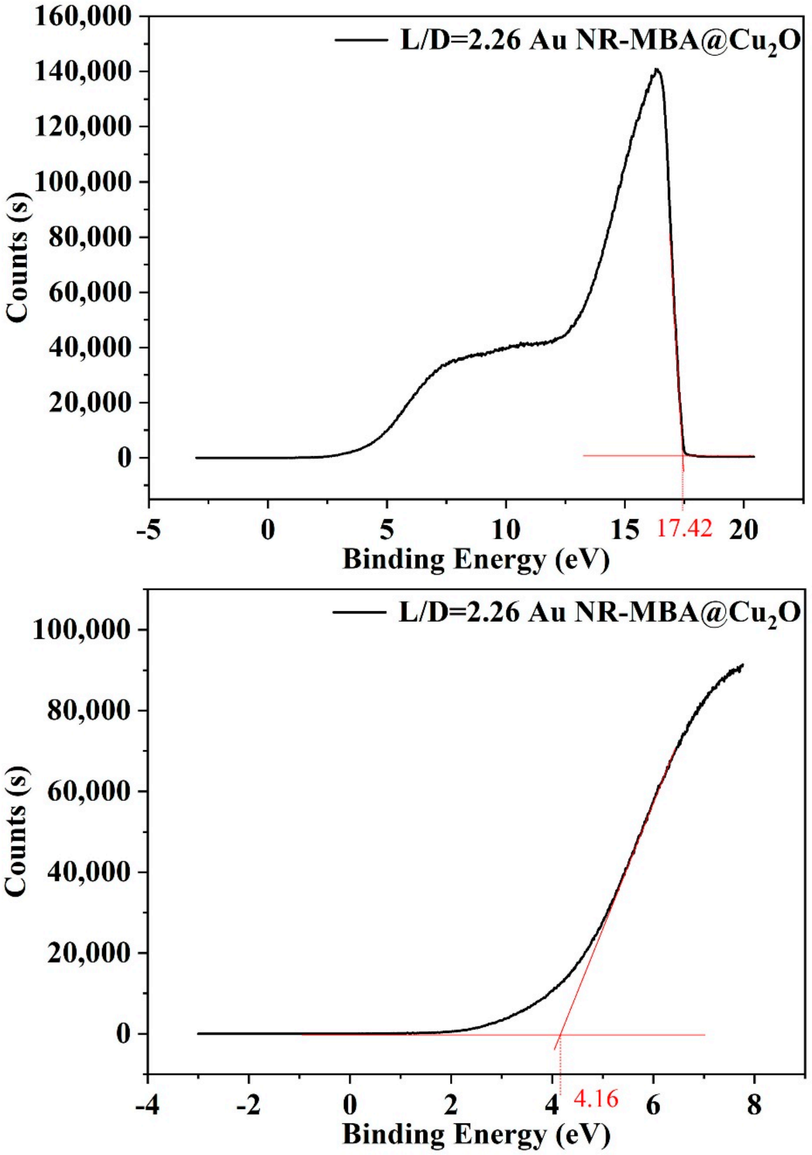

Figure S9. The UPS spectra of Au NR-MBA@Cu_2O with L/D=2.26.

According to Figure S9, the work function of Au NR-MBA@Cu_2O with L/D=2.26 is 3.8 eV, and the VB of Cu_2O is at 7.96 eV. The band gap of Cu_2O between the VB and CB is 2.2 eV. Thus, the CB of Cu_2O is at 5.76 eV.

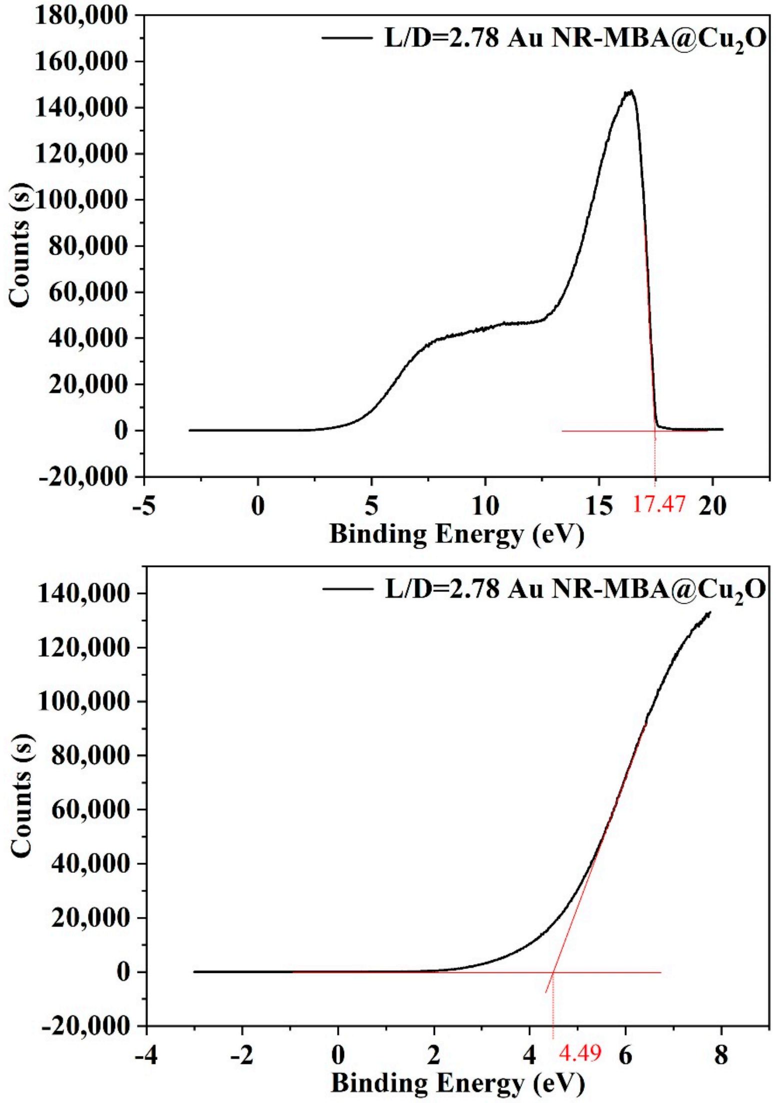

Figure 10. The UPS spectra of Au NR-MBA@Cu_2O with L/D=2.78.

According to Figure S10, the work function of Au NR-MBA@Cu_2O with L/D=2.78 is 3.75 eV, and the VB of Cu_2O is at 8.24 eV. The band gap of Cu_2O between the VB and CB is 2.2 eV. Thus, the CB of Cu_2O is at 6.04 eV.

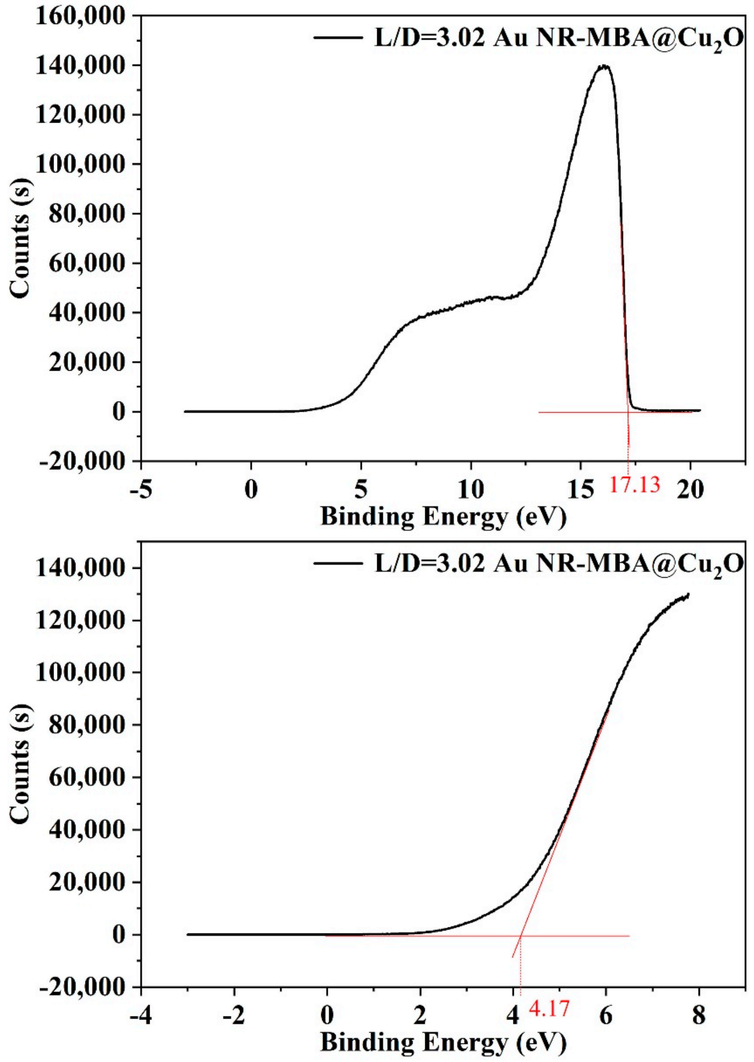

Figure S11. The UPS spectra of Au NR-MBA@Cu_2O with L/D=3.02.

According to Figure S11, the work function of Au NR-MBA@Cu_2O with L/D=3.02 is 4.09 eV, and the VB of Cu_2O is at 8.26 eV. The band gap of Cu_2O between the VB and CB is 2.2 eV. Thus, the CB of Cu_2O is at 6.06 eV.

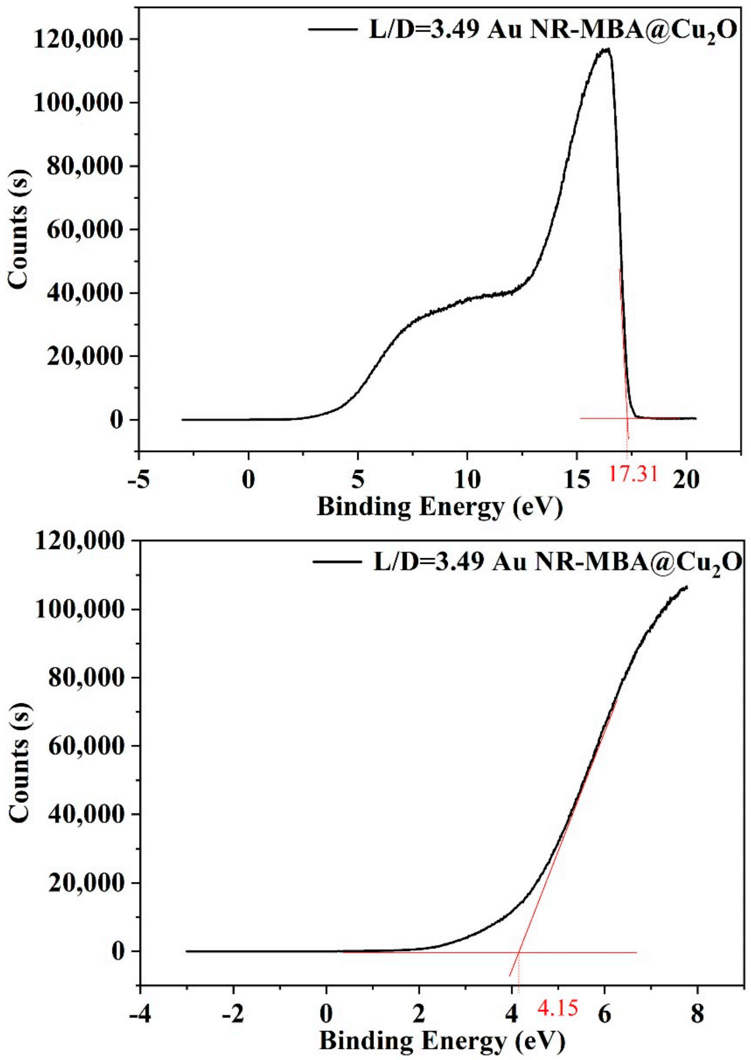

Figure S12. The UPS spectra of Au NR-MBA@Cu_2O with L/D=3.49.

According to Figure S12, the work function of Au NR-MBA@Cu_2O with L/D=3.49 is 3.91 eV, and the VB of Cu_2O is at 8.06 eV. The band gap of Cu_2O between the VB and CB is 2.2 eV. Thus, the CB of Cu_2O is at 5.86 eV.

Table S1. Specific surface area statistics for Au NR with different L/Ds.

**L/D**	**1.96**	**2.26**	**2.78**	**3.02**	3.49
S (nm^2)	4805.4	4117.42	4012.02	4081.03	3649.15
V (nm^3)	23851.04	18546.17	16767.8	16774.22	13710.01
S/V	0.2015	0.2220	0.2393	0.2433	0.2662
